# An Efficient and Selective 7‑(Diethylamino)quinolin-2(1*H*)‑One-Chalcone Fluorescent Probe for Detecting Bisulfite in Wine Samples Using a Micellar Solution

**DOI:** 10.1021/acsomega.5c00828

**Published:** 2025-06-17

**Authors:** Guillermo E. Quintero, William Tiznado, Luis Leyva-Parra, Catalina Espinoza, Oriel Sánchez-Velasco, Edwin G. Pérez, Carlos Rojas-Romo, Christian Espinosa-Bustos, Margarita E. Aliaga

**Affiliations:** † Escuela de Química, Facultad de Química y de Farmacia, 28033 Pontificia Universidad Católica de Chile, Casilla 306, Santiago 6094411, Chile; ‡ Centro de Investigación para el Diseño de Materiales (CEDEM), Departamento de Ciencias Químicas, Facultad de Ciencias Exactas, 28087 Universidad Andrés Bello República 275, 8370146 Santiago, Chile; § Centro de Investigación en Ingeniería de Materiales (CIIM), Facultad de Ingeniería y Arquitectura, Universidad Central de Chile (UCEN), Santa Isabel 1186, 8370146 Santiago, Chile; ∥ Departamento de Química, Facultad de Ciencias, 117433 Universidad de Chile, Las Palmeras 3425, Ñuñoa, Santiago 7800003, Chile; ⊥ Facultad de Química y de Farmacia, Escuela de Química y de Farmacia, Pontificia Universidad Católica de Chile, Casilla 306, Santiago 6094411, Chile

## Abstract

A new probe (*E*)-7-(diethylamino)-3-(3-(4-fluorophenyl)-3-oxoprop-1-en-1-yl)-1-methylquinolin-2­(1*H*)-one (**DQCh**) was synthesized and structurally characterized by nuclear magnetic resonance spectroscopy and HRMS. **DQCh** was photophysically characterized by employing UV–vis, fluorescence, and time-resolved fluorescence spectroscopy in combination with computational methods. This probe was studied as a turn-off fluorescent probe based on a Michael addition mechanism for sensing bisulfite (HSO_3_^–^), which is favored in the presence of an aqueous micellar solution of cetyltrimethylammonium bromide. The probe exhibited an effective and high selectivity toward bisulfite over other interfering anions with a detection limit of 0.7 μmol L^–1^. Moreover, probe **DQCh** showed great potential for its practical application in the detection of bisulfite in real samples of white wine.

## Graphical abstract

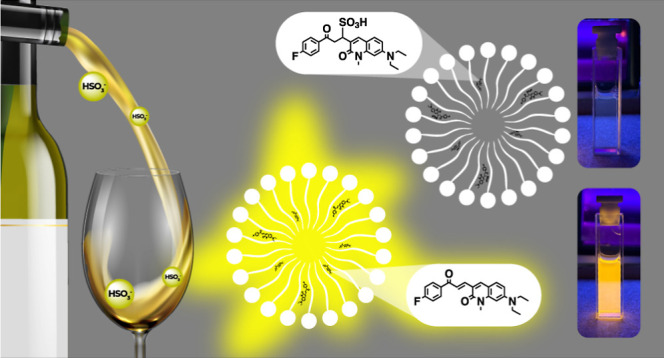

## 1 Introduction

Over the past decade, different chemistry tools have been implemented to detect and quantify relevant analytes present in biological, environmental, or chemical systems. Fluorescence is an excellent alternative due to its high sensitivity and selectivity. Therefore, the development of fluorescent probes for this purpose has risen exponentially. Different organic scaffolds have been used, including boron dipyrromethene difluoride (BODIPY),^1^ cyanines,^2^ rhodamines,^3^ fluoresceins,^4^ coumarins,^5^ quinolin-2­(1*H*)-one,^6^ etc. About the aforementioned organic scaffolds, there are currently only a limited number of studies utilizing quinolone derivatives as fluorescent probes, in stark contrast to the other scaffolds previously discussed. Compared with other cores, the insertion of quinolinones in the development of probes is very recent. It has shown a synergetic effect when included, for example, in sensors for the selective detection of Hg^2+^ in aqueous methanol media.^7^ When complexing quinolin-2­(1*H*)-ones derivatives to terbium, it is possible to detect the pesticides Malathion and Crotoxyphos,^8^ and also the insertion of the fluorophore quinolin-2­(1*H*)-one link to a Schiff base makes it possible to detect hypochlorite in cells.^9^ However, despite these efforts, there is still limited research on the photophysical properties of quinolin-2­(1*H*)-one derivatives. As mentioned before, these publications demonstrate the utility of quinolinone’s derivatives in probe design and suggest the potential for broader applications in the field of fluorescent probes. In this sense, our research has been focusing on the influence of surfactants on quinolin-2­(1*H*)-one’s derivatives, a field that has not been deeply explored with this kind of structure and could expand their applications as fluorescent probes.

Using surfactants is an excellent strategy because they can enhance solubility and modulate the photophysical properties of a specific organic molecule when used as an aqueous micellar solution over the critical micelle concentration (CMC).^10,11^ Therefore, there is evidence that surfactants such as cetyltrimethylammonium bromide (CTABr), cetylpyridinium bromide, *N*-tetradecyl-*N*,*N*-dimethyl-3-ammonio-1-propanesulfonate (SB3-14), polyethylene glycol hexadecyl ether (Brij-58), among others, increased the reactivity of probes toward different analytes.^12–15^ It is important to clarify that there is still a lack of studies examining the surfactant’s impact on the physicochemical properties of quinolin-2­(1*H*)-ones. Recently, our research group conducted a study investigating the kinetic behavior of a 6-methoxy-quinolin-2­(1*H*)-one derivative in the presence of cationic and zwitterionic surfactants with bisulfite (HSO_3_^–^) to study the reactivity toward this analyte, finding that it displays up to 200-fold rate acceleration in CTABr micellar media compared with zwitterionic media.^16^ Therefore, using this medium is relevant for developing more efficient probes that can be used under mild conditions, such as in water, and avoid toxic organic solvents. Additionally, micelles enhance the reactivity and selectivity toward analytes such as bisulfite.

In this sense, bisulfite is one of the analytes of interest, and the development of fluorescent probes focuses on detecting this analyte in food samples, such as wine (see Table S1). This is because bisulfite is used in the food industry as a preservative in certain types of food, and it has been shown that high concentrations of bisulfite present health risks.^17^ This analyte has been associated with respiratory issues, particularly asthma and allergic reactions.^18^ Therefore, its detection in food and environmental samples is crucial to public health safety. In this context, quinolin-2­(1*H*)-one-based fluorescent probes could offer an efficient and selective means of detecting and quantifying bisulfite in real samples.

In this study, we present the synthesis, structural analysis, and photophysical characterization of a novel 7-(diethylamino)-quinolin-2­(1*H*)-one chalcone-based derivative (Figure 1). Additionally, we investigated its reactivity with bisulfite in the presence of cationic CTABr and zwitterionic SB3-14 surfactants to subsequently quantify this analyte in wine samples. Notably, this work represents the first report on using surfactants to modulate the physicochemical properties and reactivity of a 7-(diethylamino)-quinolin-2­(1*H*)-one derivative. The potential applications of this probe in the detection and quantification of bisulfite in real samples, particularly in the food industry, are promising and warrant further exploration.

**Figure 1:**
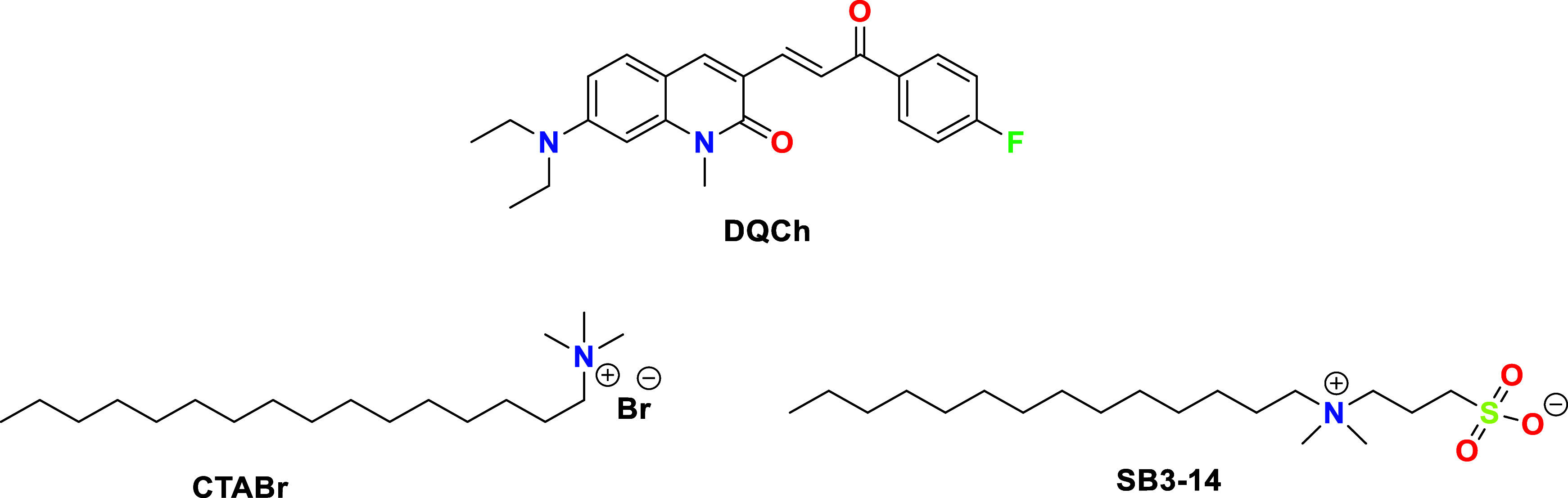
Chemical structures of dye (*E*)-7-(diethylamino)-3-(3-(4-(4-fluorophenyl)-3-oxoprop-1-en-1-yl))-1-methylquinolin-2­(1*H*)-one (**DQCh**) and the cationic cetyltrimethylammonium bromide (CTABr) and zwitterionic *N*-tetradecyl-*N*,*N*-dimethyl-3-ammonio-1-propanesulfonate (SB3-14) surfactants employed in this work.

## 2 Materials and Methods

### 2.1 Materials

All reagents and solvents, including deuterated ones, were purchased from Merck and employed as received. Column chromatography was done on silica gel (Merck, type 60, 0.063–0.2 mm). All solutions were prepared freshly.

### 2.2 Synthesis of (*E*)-7-(Diethylamino)-3-(3-(4-(4-fluorophenyl)-3-oxoprop-1-en-1-yl))-1-methylquinolin-2­(1*H*)-one (DQCh)

To a mixture of 7-(diethylamino)-1-methyl-quinolin-2­(1*H*)-one-3-carbaldehyde (0.050 g, 0.19 mmol, 1.0 equiv) with 4-fluoroacetophenone (0.036 mL, 0.23 mmol, 1.2 equiv) in H_2_O/MeOH 1:1 (2.00 mL) was added KOH (0.018 g, 0.31 mmol, 1.6 equiv). The reaction mixture was vigorously stirred at room temperature for 24 h. Then, the precipitate that formed was filtered off, washed several times with deionized water, and purified by column chromatography (AcOEt/Hexane 1.5:1) to give an orange solid (0.024 g, 32% yield). ^1^H nuclear magnetic resonance (NMR) (400 MHz, ACN-*d*_3_): δ = 8.13 (d, *J* = 15.4 Hz, 1H), 8.08 (dd, *J* = 8.9, 5.5 Hz, 2H), 8.02 (s, 1H), 7.79 (d, *J* = 15.4 Hz, 1H), 7.43 (d, *J* = 9.0 Hz, 1H), 7.24 (t, *J* = 8.9 Hz, 2H), 6.71 (dd, *J* = 9.0, 2.4 Hz, 1H), 6.38 (d, *J* = 2.4 Hz, 1H), 3.64 (s, 3H), 3.50 (q, *J* = 7.1 Hz, 4H), 1.21 (t, *J* = 7.0 Hz, 6H). ^13^C NMR (101 MHz, ACN-*d*_3_): δ = 190.50, 166.78 (d, ^1^ *J*_C–F_ = 251.0 Hz), 162.99, 152.92, 144.52, 142.82, 142.22, 132.72, 132.55 (d, ^4^ *J*_C–F_ = 8.7 Hz), 132.32 (d, ^3^ *J*_C–F_ = 9.4 Hz, 2C), 122.62, 120.24, 116.83 (d, ^2^ *J*_C–F_ = 22 Hz, 2C), 112.23, 110.34, 95.87, 46.02 (2C), 30.32, 13.25 (2C). ^19^F NMR (376 MHz, ACN-*d*_3_): δ = −109.08. HRMS *m*/*z* [M + H]^+^ calculated for C_23_H_23_FN_2_O_2_ 379.1816; found 379.1990.

### 2.32.3 Nuclear Magnetic Resonance Studies

^1^H, ^13^C, DEPT-135, and ^19^F NMR spectra were obtained at 25 °C on a Bruker Avance 400 MHz spectrometer using tetramethylsilane as the internal standard. NMR spectra were processed with MestreNova v14.2 software. All solutions were prepared by mixing appropriate volumes of **DQCh** solutions on acetonitrile (ACN)-*d*_3_ (ACN-*d*_3_) and water-*d*_2_ (D_2_O).

### 2.4 UV–vis and Fluorescence Spectra Measurements

Stock solutions of **DQCh** in dimethyl sulfoxide and surfactants in aqueous solutions were prepared daily. The absorption spectra were recorded on a Cary 60 UV–visible spectrophotometer. The fluorescence spectra were obtained by using a Cary eclipse fluorescence spectrophotometer.

### 2.5 Fluorescence Emission Quantum Yields

The fluorescence quantum yield of **DQCh** was measured using coumarin 153 in EtOH as standard (ϕ_s_ = 0.53)^19^ according to eq 1:^13^

1 $$\phi_{x}=\phi_{s}\left(\frac{{grad}_{x}}{{grad}_{s}}\right)\left(\frac{n_{x}^{2}}{n_{s}^{2}}\right)$$

where ϕ is the quantum fluorescent yield, subscripts x and s indicate the sample (**DQCh** solutions in this case) and standard, respectively, *n* is the refractive index, and Grad is the slope from the plot of integrated fluorescence intensity versus absorbance.

### 2.6 Binding Constant of DQCh with CTABr and SB3-14 Surfactants

The binding constants (*K*_s_) were obtained by fluorescence spectroscopy exciting at the maximum absorption wavelength of **DQCh** at a concentration of 2.0 μmol L^–1^. The value of *K*_s_ has been determined using the nonlinear regression method (eq 2)^20^

2 $$I=\frac{I_{w}^{0}+I_{m}^{0}K_{s}\left(C_{s}-CMC\right)}{1+K_{s}\left(C_{s}-CMC\right)}$$

where *I* is the fluorescence intensity of **DQCh** in the presence of surfactants, subscripts w and m indicate aqueous and micellar pseudophases, respectively, *I*_w_^0^ and I_m_^0^ correspond to the respective intensity of **DQCh** in the aqueous and micellar pseudophases, *C*_s_ is the total surfactant concentration, and CMC is the critical micellar concentration being 0.96 mM and 0.22 mM for CTABr and SB3-14, respectively.^16^

### 2.7 Time-Resolved Fluorescence Measurements

Fluorescence decays were measured using a Lifespec II picosecond fluorescence lifetime spectrometer from Edinburgh Instruments. The experimental conditions used in this work were carried out according to the report by Zuñiga-Núñez et al.^21^ The values were obtained using an excitation source of a 458 nm laser diode (EPL-458, with an fwhm of 100 ps). The maximum number of counts collected to determine fluorescence lifetimes was 10,000 counts. The emission was collected at wavelengths corresponding to the fluorescence maximum of **DQCh** in different solvents. The IRF was deconvoluted with fluorescence decays to obtain fluorescence lifetimes.

### 2.8 DFT Calculations

The geometric optimization of the **DQCh** molecule in its ground state was carried out using DFT with the PBE0 functional^22^ and the Def2-TZVP basis set,^23^ a reliable combination for accurately predicting the geometric structures of organic molecules,^24^ as demostrated in previous studies.^16^ The UV–vis spectra of **DQCh** were assessed using Time-Dependent DFT (TD-DFT)^25^ at the same theoretical level, focusing on the first 30 excited states (TD = (nstates = 30)). Solvent effects were included via the Polarizable Continuum Model (PCM).^26^ All calculations were performed using the Gaussian 16 software (revision B.01).^27^

Electrostatic surface potential (ESP) maps of the optimized structures were calculated and visualized using Multiwfn 3.3.6 ^28^ and VMD^29^ programs. Plotted at an isovalue of 0.002 au, these maps offer a detailed representation of the molecular electrostatic potential distribution. Local reactivity was analyzed using the Fukui function (FF) and evaluated through its topological analysis following an approach analogous to Bader’s method for electron density.^30^ The analysis was conducted at the same level of theory as the geometry optimizations to ensure methodological consistency and reliability. The TAFF pipeline^31^ automated the topological analysis by extracting data from *.fchk files and compiling key outputs, including atomic coordinates, attractor positions, and basin-condensed values. These computational techniques provide a detailed characterization of the structural and electronic properties of **DQCh**, complementing experimental results and contributing to understanding its physicochemical behavior.

### 2.9 Selectivity and Competition Study

All the testing anions (HSO_3_^–^, F^–^, Cl^–^, I^–^, CH_3_COO^–^, HCO_3_^–^, NO_2_^–^, NO_3_^–^, N_3_^–^, IO_4_^–^, SO_4_^2–^, S_2_O_3_^2–^, and S_2_O_8_^2–^) were prepared from their sodium salts. The anion HS^–^ was prepared from a potassium salt. The solution of cysteine (Cys), homocysteine (Hcy), and glutathione (GSH) were prepared freshly in Milli-Q water. The selectivity and competition studies were conducted by adding 50 equiv of interfering ions to a 1.8 μmol L^–1^ **DQCh** solution. Both experiments were carried out in an aqueous micellar solution of CTABr (10 mM), 1% DMSO, buffer PBS (10 mM) at pH 7.4, 27 °C, and λ_ex_ = 464 nm with slit 5 nm/5 nm.

### 2.10 Determination of the Limit of Detection and Quantification

The limit of detection (LOD) and limit of quantification (LOQ) were calculated based on the fluorescence of the successive bisulfite addition. LOD and LOQ were calculated using eqs 3 and 4:^32^

3 $$LOD=\frac{3.3\sigma }{m}$$

4 $$LOQ=\frac{10\sigma }{m}$$

where σ is the standard deviation of the intercept and *m* is the slope of fluorescence intensity versus bisulfite concentration.

### 2.11 Preparation of Real Samples

The method used in this work was carried out according to Gómez et al.^33^ with some variations. Each sample was added to the fluorescence cuvette, containing 2.5 mL of phosphate buffer solution (10 mM, pH 7.4, 1% v/v DMSO) in the presence of 10 mM of CTABr. The sample concentration was determined by the fluorometric method reported in this work by adding an aliquot into the cuvette between 15 and 30 μL of wine samples. Three white wines were analyzed, and the bisulfite concentration was determined. The wine samples were obtained from local markets.

## 3 Results and Discussion

### 3.1 Synthesis and Structural Characterization of DQCh

The design principle of a fluorescent probe for bisulfite detection is related to the nucleophilic nature of the analyte. Thus, we proposed an α,β-unsaturated carbonyl compound as the Michael acceptor, with 7-(diethylamino)­quinolin-2­(1*H*)-one serving as the fluorophore. The synthesis of the **DQCh** probe is summarized in Scheme 1. The precursor, 7-(diethylamino)­quinolin-2­(1*H*)-one-3-carbaldehyde, was synthesized according to the methodology reported by Samaan et al.^34^ and Singh et al.^35^ The novel **DQCh** compound was synthesized by coupling 7-(diethylamino)-1-methyl-quinolin-2­(1*H*)-one-3-carbaldehyde (**4**) with 4-fluoroacetophenone, yielding 32%. Structural characterization was performed using ^1^H, ^13^C, and ^19^F NMR and HRMS spectroscopy (Figures S1–S4). The coupling reaction was confirmed by the presence of α,β-unsaturated carbonyl, whose proton chemical shifts were observed at 7.79 ppm (H_α_) and 8.13 ppm (H_β_). The ^1^H NMR spectra confirmed the trans-isomer of **DQCh** based on the coupling constants (*J*) for the α,β protons of the chalcone moiety, with a value of 15.4 Hz, consistent with literature reports.^36^

**Scheme 1:**
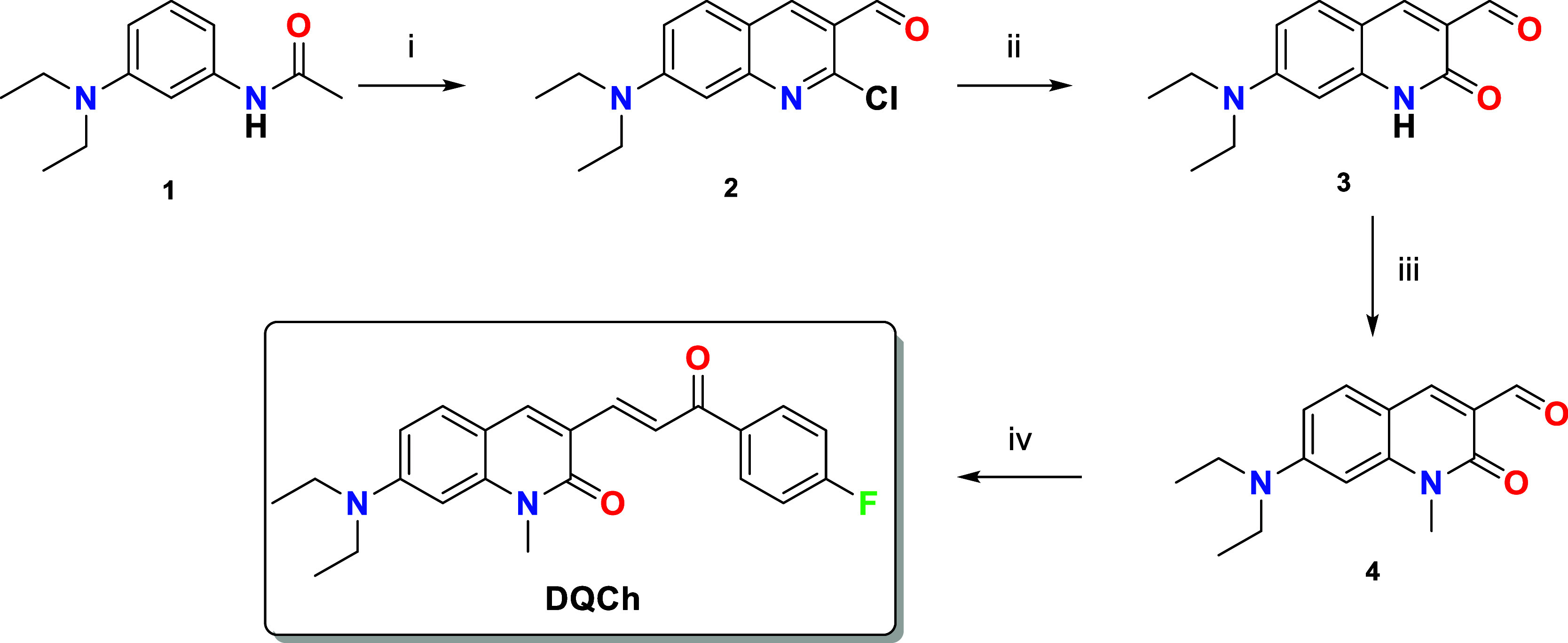
Methodology for **DQCh** Formation. Reagents and Conditions: (i) POCl_3_/DMF, 20 min, 50 °C; (ii) Acetic Acid 70%, 4 h, Reflux; (iii) NaH, MeI, DMF, 2 h, 0 °C; and (iv) 4-Fluoroacetophenone, KOH, MeOH, 24 h

### 3.2 Photophysical Characterization

In order to understand its behavior in different media, the photophysical properties of **DQCh** were evaluated in common solvents, including ethyl acetate (AcOEt), tetrahydrofuran (THF), dichloromethane (DCM), ACN, dimethyl sulfoxide (DMSO), ethanol (EtOH), and water, as shown in Figure S5. Table 1 summarizes all of the photophysical properties, such as the maxima wavelength on UV–VIS and fluorescence (λ_abs_ and λ_em_), quantum fluorescent yields (ϕ_f_), and fluorescence lifetimes (τ). The absorption behavior of **DQCh** in these solvents showed a slight spectral variation of ±20 nm. However, a strong shift in emission was observed from an apolar solvent (AcOEt) to a protic polar solvent like water, exhibiting a strong bathochromic shift of approximately 100 nm, indicating a large value of the dipolar moment in the excited state. This shift can also be attributed to polar solvents’ stabilization of the intramolecular charge transfer state (ICT).^37–39^ Furthermore, ϕ_f_ was calculated by using eq 1, demonstrating a notable solvent dependence.

**Table 1:** Photophysical Properties of **DQCh** in Solvents of Different Polarities

solvent	λ_abs_ (nm)	λ_em_ (nm)	ϕ_f_	τ_1_ (ns)/*A*_1_	τ_2_ (ns)/*A*_2_	τ_3_ (ns)/*A*_3_	χ^2^
AcOEt	444	501	0.711		0.67(18%)	1.79(82%)	1.108
THF	450	513	0.602		0.85(1%)	1.89(99%)	1.039
DCM	451	523	0.600		0.62(10%)	1.94(90%)	1.134
ACN	450	545	0.509		0.52(6%)	2.34(94%)	1.149
DMSO	464	560	0.587		1.00(1%)	2.54(99%)	1.194
EtOH	449	579	0.310	0.01(78%)	1.59(18%)	2.71(4%)	1.031
water	449	609	0.010	n.d^a^	n.d	n.d	n.d

a Not detected.

**DQCh** exhibits the lowest fluorescent quantum yield in an aqueous solution compared to that of the other solvents used in this study. This is attributable to the formation of a twisted intramolecular charge transfer (TICT) state favored by an increased solvent polarity and also the protic nature of the solvents.^40–42^ Specifically, **DQCh** presents a site susceptible to promoting TICT from the diethylamino donor group (−NEt_2_). This substituent has been widely studied for its ability to induce intramolecular torsions.^43–46^ This leads to a nonemissive TICT state and a reduced contribution from the ICT state, resulting in a low quantum fluorescent yield in protic polar solvents. In contrast, **DQCh** displayed higher ϕ_*f*_ values for the other solvents than EtOH and water. This effect is favored due to a decrease in the TICT formation rate with increasing solvent viscosity and a reduction in solvent polarity, promoting an emissive ICT state.^47^ Additionally, the fluorescent lifetime of the quinoline-2­(1*H*)-one derivative was measured in the solvents mentioned above (see Table 1) to determine the contribution to the fluorescence ICT, TICT, and locally excited (LE) states. In protic polar solvents such as EtOH, **DQCh** presented three fluorescent lifetimes, with the first one contributing the most and showing the shortest lifetime (τ_1_ = 0.01 ns, *A*_1_ = 78%), consistent with the TICT state, which undergoes rapid nonemissive deactivation.^21^ Moreover, the longer fluorescent lifetimes, τ_2_ (1.59 ns) and τ_3_ (2.71 ns), are related to emissive deactivations of LE and ITC states, respectively.^21,40,48^ In apolar and polar aprotic solvents (ACN and DMSO), the fluorescence emission of **DQCh** shows two fluorescent lifetimes (τ_2_ and τ_3_) with no formation of TICT exited state observed.

It is important to note that fluorescence lifetimes could not be obtained in water, as shown in Table 1. The absence of fluorescence lifetimes, along with low fluorescence quantum yields, may be attributed to **DQCh** forming aggregates in water due to its low solubility. The formation of aggregates in water has already been extensively studied in similar molecules, such as coumarins.^49,50^ This observation is significant and warrants further discussion in future studies.

#### 3.2.1 Micellar Influence on Photophysical Properties

Several studies have reported using aqueous micellar solutions (containing cationic and zwitterionic surfactants) that can induce changes in the photophysical properties of compounds.^11,13,51–54^ In this context, it is valuable to understand how the photophysical properties of this novel quinolin-2­(1*H*)-one derivative (**DQCh**) can be modulated by using an aqueous micellar solution. Table 2 summarizes the photophysical properties of **DQCh** in CTABr and SB3-14 micellar solution and compares them with those in water. In the absence of a surfactant, **DQCh** exhibits absorbance and fluorescence emission maxima at 449 and 609 nm, respectively, with a low quantum fluorescence yield. However, in the presence of an aqueous micellar solution, a bathochromic shift in absorbance and a blue shift in fluorescence were observed. The blue shift in emission is likely due to the apolar microenvironment around **DQCh**, which differs from that in water.^54^ Furthermore, ϕ_f_ increased 13-fold in CTABr and 20-fold in SB3-14, attributed to the higher viscosity of the Stern layer compared to the neat water. This higher viscosity of the solvent restricts the rotation of the—NEt_2_ group and, consequently, inhibits the TICT state.^11,55^

**Table 2:** Photophysical Properties of **DQCh** in Water and Aqueous Micellar Solutions

solvent^a^	λ_abs_ (nm)	λ_em_ (nm)	ε^b^(10^4^ M^–1^ cm^–1^)	ϕ_*f*_	*K*_s_^c^ (10^4^ M^–1^)	τ_1_ (ns)/*A*_1_	τ_2_ (ns)/*A*_2_	⟨τ⟩
water	449	609	2.96 ± 0.008	0.01		n.d	n.d	
CTABr	464	587	4.21 ± 0.005	0.13	3.58 ± 0.29	0.53(52%)	1.33(48%)	0.92
SB3-14	463	577	2.80 ± 0.003	0.20	0.99 ± 0.01	0.91(48%)	1.84(52%)	1.39

a Concentration of CTABr and SB3-14 used was 10 mM and 2.20 mM, respectively.

b Values obtained at λ_max._ of absorption at 25 °C.

c Values obtained by fluorescence excitation at λ_max_ of each solvent at 27 and 25 °C to CTABr and SB3-14, respectively.

Additionally, fluorescence lifetimes were measured for both surfactants that exhibited two lifetimes, with the first one (τ < 1 ns) being attributable to the LE emissive state and the second one (τ > 1 ns) corresponding to the ITC state. According to the literature, highly polar protic solvents like water are expected to exhibit a lifetime associated with the TICT state, with a predominant contribution.^41,42^ However, when an aqueous micellar solution is present, two distinct fluorescence lifetimes are observed rather than one linked to the TICT state. Unfortunately, the values cannot be compared with those in water, as **DQCh** did not exhibit fluorescence lifetimes suitable for proper reconvolution in this medium. Despite this limitation, the decay time values obtained in the micellar solution can be compared to those in other organic solvents, showing similar values. While it has been noted that a micellar environment can influence photophysical properties, it can also lead to other significant effects. Notably, in the presence of an aqueous micellar solution, **DQCh** demonstrated improved solubility compared to water, as no precipitates were observed at concentrations higher than 10 μM, and its stability in this medium was enhanced. These results highlight the importance of using a micellar medium to prevent the previously mentioned issues.

### 3.3 Binding Constants Determination by Fluorescence Study

On the other hand, we determined the binding constant (*K*_s_) of **DQCh**•CTABr and **DQCh**•SB3-14. This analysis was carried out by fluorescence spectroscopy, where the fluorescence intensity of **DQCh** increases when the micellar concentration increases, as shown in Figure 2. The presence of a micellar aqueous solution of CTABr (Figure 2a) shows a fluorescence intensity higher than that of SB3-14 (Figure 2b) when [Cs-CMC] is equal to zero. The relationship between fluorescence intensity and micellar concentration was fitting according to eq 2, and the results are summarized in Table 2.

**Figure 2:**
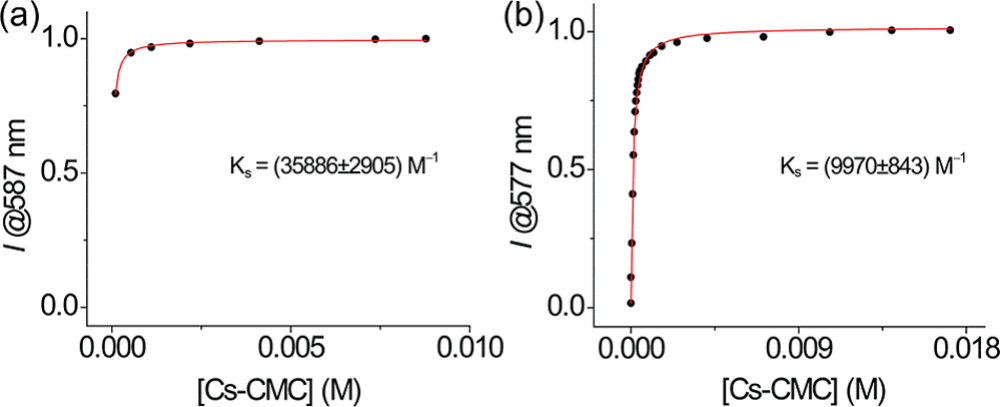
Fitting the fluorescence intensity normalized values to determine the probe–micelle association constant for **DQCh** in (a) CTABr and (b) SB3-14 aqueous micellar solution.

The K_s_ value using the CTABr surfactant was approximately 3.6-fold higher, indicating a stronger interaction than the zwitterionic surfactant. The *K*_s_ values in the order of 10^3^–10^4^ M^–1^ demonstrate the high interaction of the quinolin-2­(1*H*)-one derivative with the micelles under study, offering a rather apolar microenvironment different from neat water,^54^ confirming the hydrophobic nature of **DQCh** when this is incorporated in the Stern layer. Similar *K*_s_ orders have been reported in the literature for other dyes in an aqueous micellar solution of CTABr and SB3-14.^10,54,56^ Notably, this is the first time that *K*_s_ values for 7-(diethylamino)­quinolin-2­(1*H*)-one derivative in micelles environments are reported. Comparable *K*_s_ values were reported by Sinha and Seth,^57^ who observed *K*_s_ values on the order of 10^4^ M^–1^ for coumarin derivatives with CTABr surfactant. The high *K*_s_ value and the enhanced ϕ_f_ of **DQCh** in the presence of an aqueous micellar solution suggest the probe is fully incorporated into the micelle, preventing the presence of a free probe that could interfere with the analytical signal, additionally suggesting the potential use of this system as a probe for detecting and quantifying analytes, such as bisulfite, for analytical applications.

### 3.4 DFT Calculations

Time-dependent DFT (TD-DFT) is particularly effective in predicting UV–vis spectra among computational techniques.^58–60^ Figure 3 presents the results of TD-DFT calculations, which confirm that the dominant band corresponds to an π→π* transition. This result is significant because it underlines the electronic nature of the transitions observed in the UV–vis spectra of the **DQCh** compound. Figure 3 also illustrates a bathochromic shift attributed to the solvent effect. This effect was modeled using the implicit solvent approximation of the PCM.

**Figure 3:**
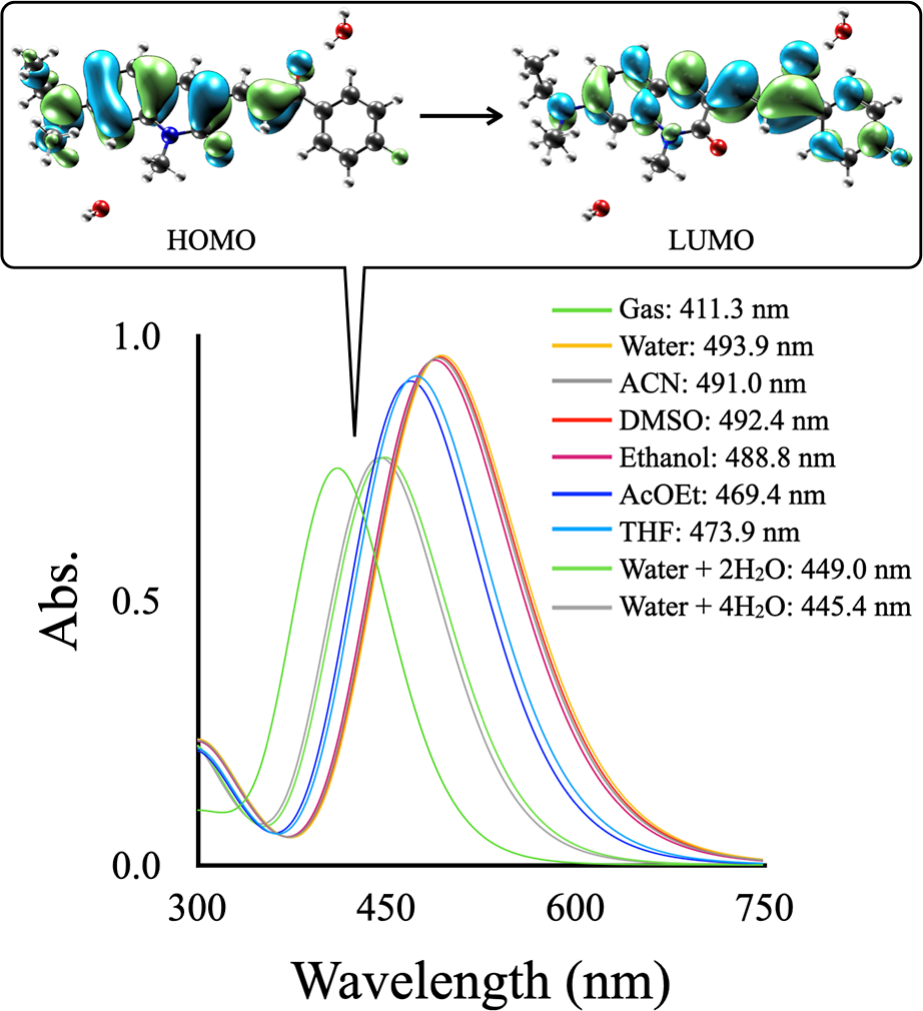
Computational UV–vis spectra of **DQCh** in various solvents.

Furthermore, the influence of explicit water molecules in the surrounding region of the **DQCh** was assessed by incorporating between two and four solvent molecules within the regions exhibiting maximal and minimal electrostatic potential (Figure 4).

**Figure 4:**
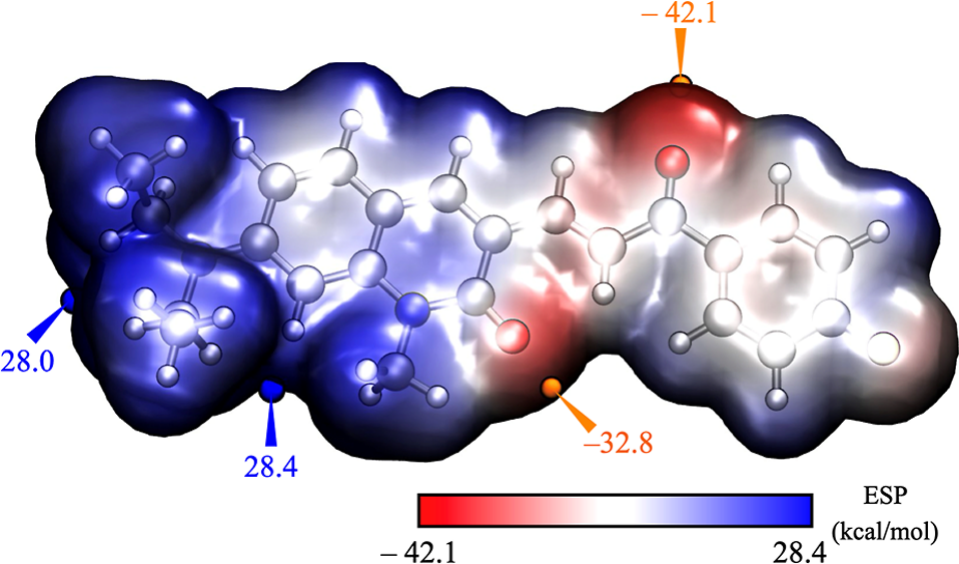
ESP distribution maps of **DQCh**, with electron density set at 0.002 au, showing maximum (blue) and minimum (orange) potential values.

The explicit inclusion of two water molecules resulted in an absorption wavelength maximum of 449 nm, which is in excellent agreement with the experimental data. However, a slight shift to 445.4 nm was observed when four water molecules were added, indicating that adding more solvent molecules does not significantly impact. The PCM model qualitatively captures the bathochromic shifts observed experimentally, reflecting the influence of the solvent. These results indicate that PCM is adequate for describing the solvent effects involved. Additionally, preliminary theoretical insights into the regioselectivity of the addition reaction were obtained through the analysis of the FF, as illustrated in Figure 5. The FF, calculated using two approaches (finite-difference and orbital-weighted),^61–64^ consistently identified the unsaturated carbon adjacent to the quinolinone ring as the most reactive site for nucleophilic attack. It is noteworthy that, although Fukui’s function also points to the oxygen in the carbonyl group as a reactive center, its reactivity is mainly related to electrostatic interactions rather than direct involvement in bond-forming processes.

**Figure 5:**
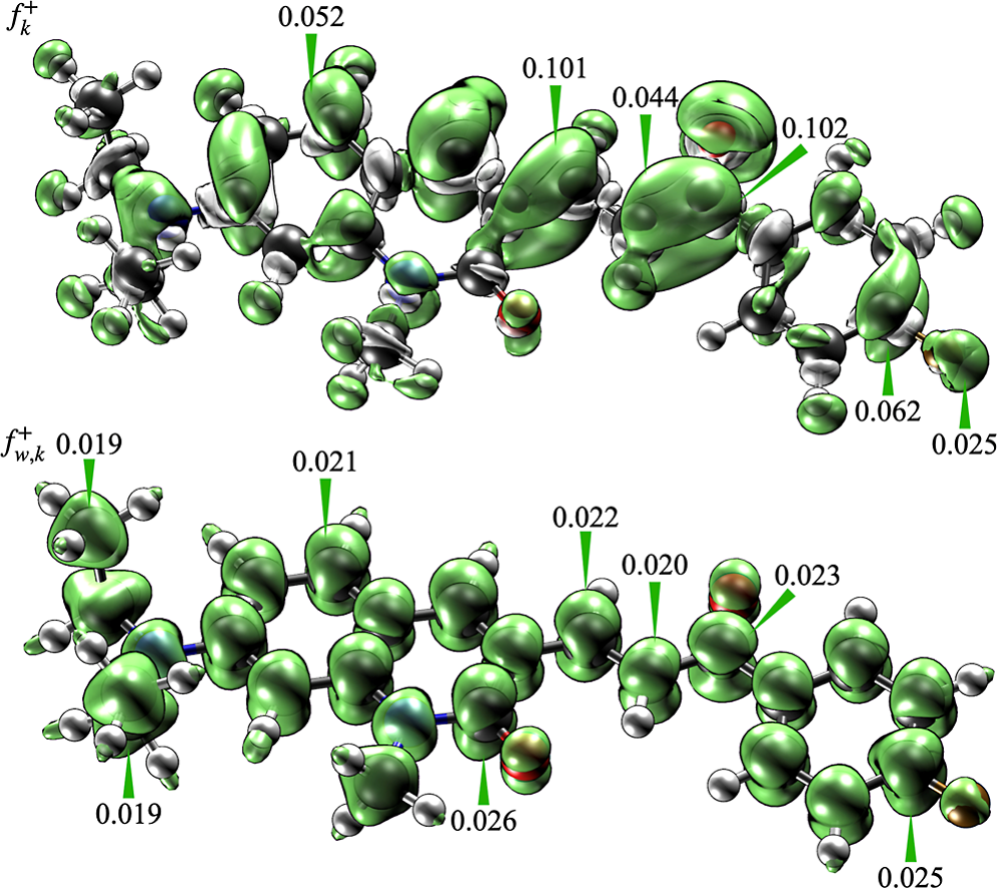
Computed Fukui function for the **DQCh** molecule using two different methods—finite differences (*f*_k_^+^) and orbital-weighted (*f*_w,k_^+^)—specifically highlighting regions with the highest reactivity toward nucleophilic attacks.

### 3.5 Bisulfite Effect on Photophysical Properties

#### 3.5.1 Bisulfite Effect on Micellar Medium

After carrying out experimental studies on the photophysical behavior of **DQCh** in different solvents with varying polarities, including micellar systems, and in conjunction with theoretical studies that determined the sites susceptible to nucleophilic attack, the photophysical response of the quinolin-2­(1*H*)-one derivative toward HSO_3_^–^ was evaluated. Initially, this response was assessed in an aqueous micellar solution of CTABr (10 mM) in PBS (10 mM, pH 7.4) at 27 °C. Figure 6a illustrates the time-dependent changes in the absorbance and fluorescence of **DQCh** in the presence of bisulfite. Within 3 h, the absorbance band at 464 nm progressively decreased, while a new band emerged at 380 nm, accompanied by noticeable colorlessness. Additionally, an isosbestic point at 400 nm indicated the formation of a new compound.

**Figure 6:**
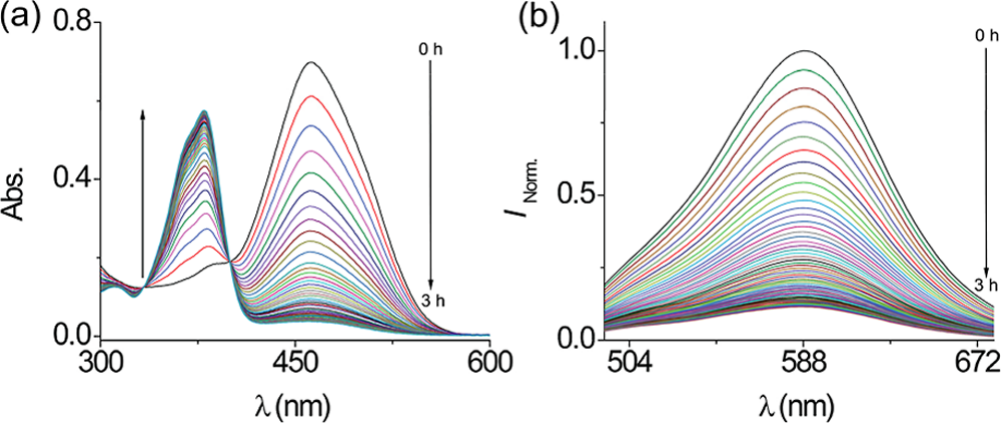
Time-dependent (0–3 h) (a) absorption spectrum of **DQCh** (26 μM) and (b) emission spectrum of **DQCh** (3 μM) in the presence of 50 equiv of HSO_3_^–^ in CTABr (10 mM) and PBS (10 mM) at pH 7.4 and 27 °C. λ_ex_ = 464 nm; slit, 5 nm/5 nm.

Figure 6b shows a similar trend in fluorescence, where the emission band at 590 nm decreased over time. Interestingly, when the reaction was conducted using the SB3-14 surfactant, minimal changes in both absorbance and fluorescence were observed (Figure S6). Complementary, ^1^H NMR studies confirmed the weak interaction between bisulfite and the SB3-14 surfactant (see Figure S7), in contrast to the stronger interaction observed with CTABr, which exhibited a higher chemical shift of the proton of the head surfactant (see Figure S8).

These findings align with our group’s previous study, which investigated a 6-methoxy-quinolin-2­(1*H*)-one derivative with an isoxazole moiety. We reported that the Michael-type reaction exhibited low rate values in the presence of sulfobetaine-type zwitterionic surfactants (SB3-n, *n* = 10, 14, and 16), likely due to the low adsorption rates of HSO_3_^–^ ions on the surfactant headgroup.^16^ Based on these results, CTABr was identified as the optimal surfactant, demonstrating the most pronounced photophysical response to bisulfite. Consequently, all subsequent experiments were conducted using an aqueous micellar solution of CTABr.

#### 3.5.2 Evidence of Michael Adduct Formation

As shown in Figure 7a,b, the addition of bisulfite to **DQCh** via the Michael reaction was confirmed by ^1^H NMR. The partial ^1^H NMR spectrum displays the isolated derivative before and after adding HSO_3_^–^ (**DQCh**-SO_3_H). The signals of the H_α_ and H_β_ protons corresponding to the carbon–carbon double bond located between 8.51 and 8.41 ppm present a high-field shift. Notably, the H_β_ proton shifts to 5.47 ppm, while the H_α_ could not be clearly identified due to the overlap with the ACN signals. Another proton that presented a shift to a high field was H_δ_ from carbon δ, carbon adjacent to carbon β, which initially appeared at 8.66 ppm and shifted to 8.19 ppm. These changes confirm the bisulfite addition to the double bound. Similar shifts have been reported for other SO_2_ derivatives probes under comparable conditions.^33,65,66^ The evidence demonstrates that **DQCh** is a suitable probe for bisulfite, and it is effective in generating a Michael adduct product in the presence of bisulfite (see Scheme 2).

**Figure 7:**
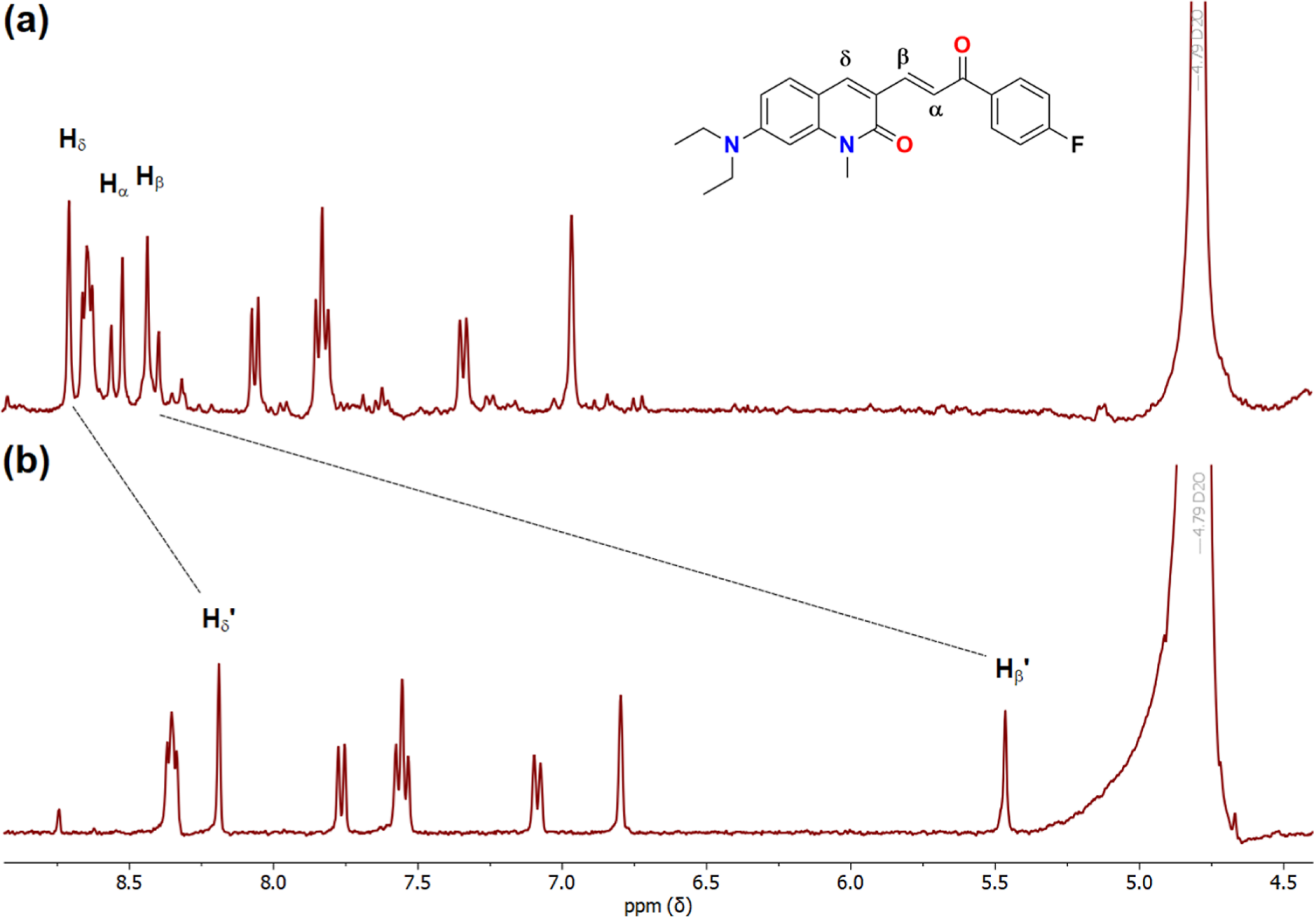
Partial ^1^H NMR of (a) **DQCh** and (b) **DQCh**-SO_3_H in CH_3_CN/D_2_O (1:2).

**Scheme 2:**
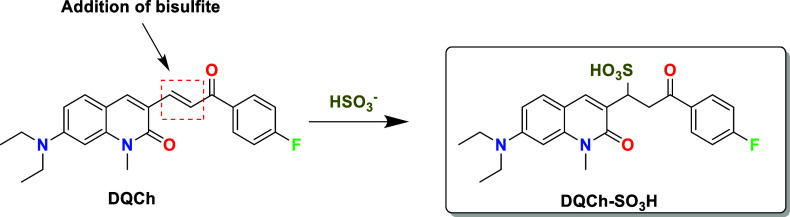
Proposed Reaction of **DQCh** with Bisulfite

### 3.6 Selectivity and Competition Studies of DQCh toward SO_2_ Derivatives and Other Species

It is well-known that chalcone derivative dyes are used as sensors for a wide variety of biothiols, anions, and cations.^65,67–71^ Therefore, it is interesting to evaluate the selectivity of this new quinolin-2­(1*H*)-one derivative chalcone-based toward bisulfite. To evaluate the selectivity, we investigate the fluorescence response of **DQCh** in the presence of several common interfering anions, such as F^–^, Cl^–^, I^–^, CH_3_COO^–^, HCO_3_^–^, NO_2_^–^, NO_3_^–^, N_3_^–^, IO_4_^–^, HS^–^, SO_4_^2–^, S_2_O_3_^2–^, S_2_O_8_^2–^, and biothiols (i.e., cysteine (Cys), homocysteine (Hcy), and glutathione (GSH)) in CTABr-PBS (1% v/v DMSO) at 27 °C. Figure 8a displays the spectral variation of the emission band in the presence of all anions. Figure 8b shows the changes in the emission intensity at 590 nm. It is possible to appreciate that most of the possible interferents did not induce any significant change in emission. We observed that **DQCh** displayed a slight variation of its emission when exposed to S_2_O_8_^2–^ and GSH but showed a substantial reduction in fluorescence intensity upon the addition of HSO_3_^–^ and IO_4_^–^.

**Figure 8:**
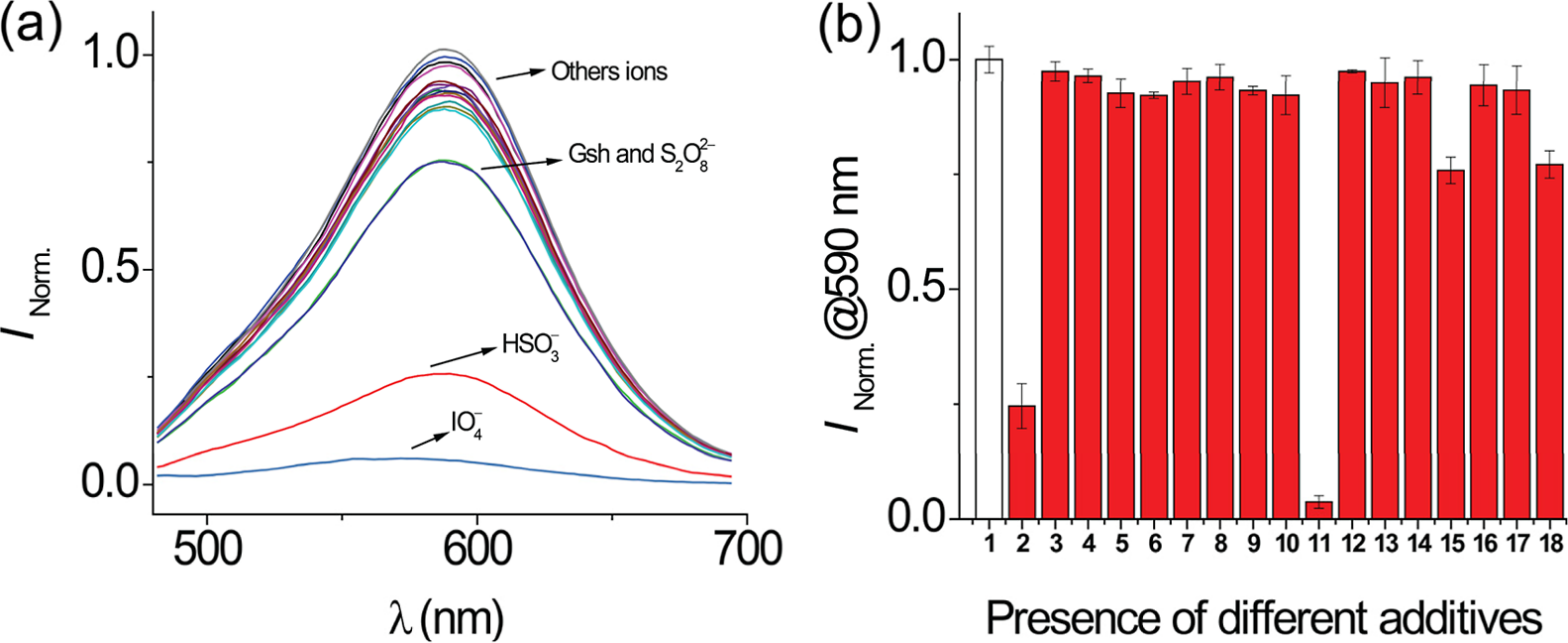
(a) Fluorescence spectrum of **DQCh** (1.8 μM) upon addition of various anions (100 μM). (b) Fluorescence response of **DQCh** at 590 nm. The white bar represents **DQCh** alone (1) and the red bar represents HSO_3_^–^ (2); F^–^ (3); Cl^–^ (4); I^–^ (5); CH_3_COO^–^ (6); HCO_3_^–^ (7); NO_2_^–^ (8); NO_3_^–^ (9); N_3_^–^ (10); IO_4_^–^ (11); HS^–^ (12); SO_4_^2–^ (13); S_2_O_3_^2–^ (14); S_2_O_8_^2–^ (15); Cys (16); Hcy (17); and GSH (18). CTABr (10 mM), 1% v/v DMSO, buffer PBS (10 mM), pH 7.4, and 27 °C. λ_ex_ = 464 nm; slit, 5 nm/5 nm.

On the other hand, a competitive analysis of various anions with HSO_3_^–^ was carried out. Figure 9 illustrates no significant variation in the fluorescence intensity of **DQCh** at 590 nm upon the addition of 50 equiv of interfering anions. However, a notable variation was presented in the presence of IO_4_^–^ and, to a lesser degree, with S_2_O_8_^2–^. A significant variation (in the presence of IO_4_^–^) could be generated due to the reaction between bisulfite and periodate.^72^

**Figure 9:**
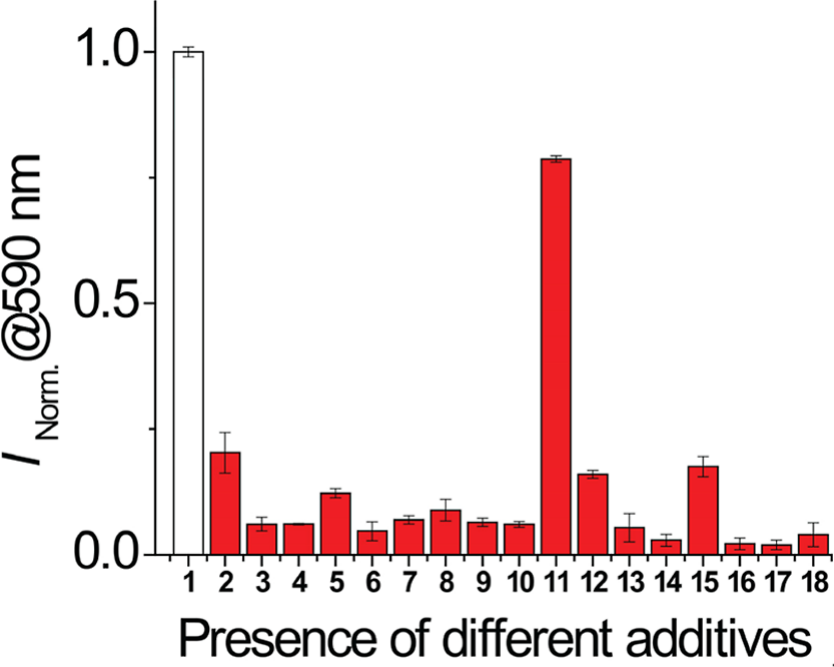
Fluorescence response of **DQCh** (1.8 μM) at 590 nm upon addition of various anions (100 μM). The white bar represents **DQCh** alone (1) and red bars represent **DQCh** in the presence of HSO_3_^–^ (2) and other anions: F^–^ (3); Cl^–^ (4); I^–^ (5); CH_3_COO^–^ (6); HCO_3_^–^ (7); NO_2_^–^ (8); NO_3_^–^ (9); N_3_^–^ (10); IO_4_^–^ (11); HS^–^ (12); SO_4_^2–^ (13); S_2_O_3_^2–^ (14); S_2_O_8_^2–^ (15); Cys (16); Hcy (17); and GSH (18). CTABr (10 mM), 1% v/v DMSO, buffer PBS (10 mM) pH 7.4, and 27 °C. λ_ex_ = 464 nm; slit, 5 nm/5 nm.

### 3.7 Determination of Analytical Parameters

The analytical parameters of the method were evaluated by the construction of a calibration plot (Figure 10). Different solutions with variable concentrations of bisulfite (1.8–300.0 μmol L^–1^) in a CTABr-PBS (1% v/v DMSO, pH 7.4) and 1.8 μmol L^–1^ **DQCh** were prepared. The fluorescence intensity of **DQCh** was measured at 590 nm over a period of 0 h (*I*_0_) to 3 h (*I*_f_) for each solution at 27 °C. Three sets of solutions were prepared to construct three calibration plots. The difference between (*I*_0_) and (*I*_f_) was used as the analytical response. The calibration plot showed a linear response until a bisulfite concentration of 47.9 μmol L^–1^. The detection and quantification limits were 0.7 and 2.1 μmol L^–1^, respectively. Therefore, the linear range of the method was 2.1–47.9 μmol L^–1^.

**Figure 10:**
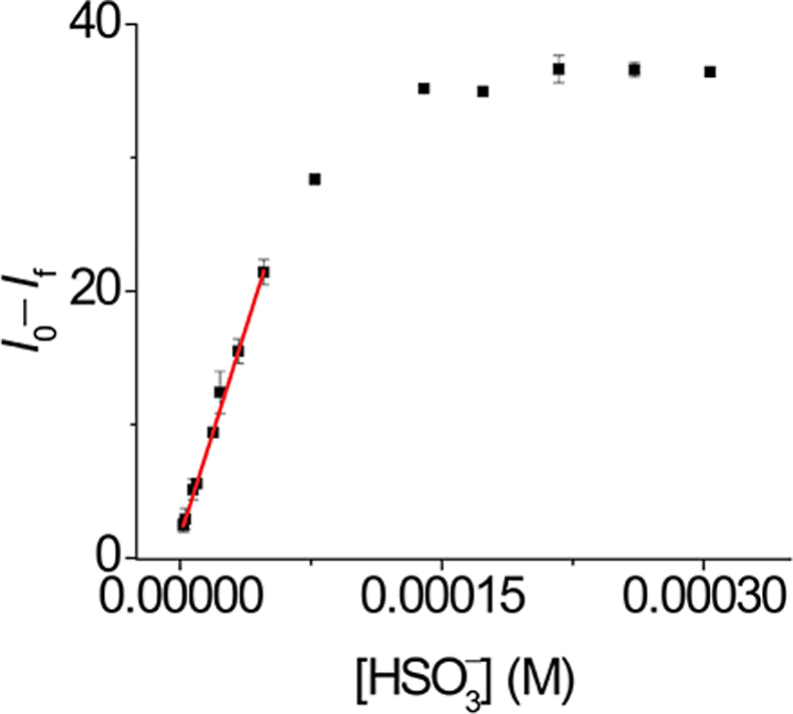
Calibration plot for bisulfite determination using *I*_0_–*I*_f_ as analytical response in a bisulfite concentration range between 1.8 and 300 μmol L^–1^. The results were reported as the mean ± standard deviation of triplicate experiments and can be fitted by a linear regression equation *I*_0_–*I*_f_ = (414,506 ± 7643) [HSO_3_^–^] mol L^–1^ + (1.63 ± 0.087) with *R* ^2^ = 0.9976.

The relatively low LOD value of 0.7 μmol L^–1^ for **DQCh** underscores its high sensitivity to the target analytes, making it a promising candidate for probe applications. This level of sensitivity is particularly advantageous for detecting trace amounts of compounds in complex environments. Notably, the observed LOD is comparable to those of other fluorescent probes reported in the literature (see Table S1), further demonstrating the effectiveness of **DQCh** as a sensitive fluorescent probe in a mild medium.

### 3.8 Real Samples Analysis

To demonstrate the applicability of the fluorescent probe, **DQCh** + CTABr was evaluated for bisulfite determination in real samples. To perform this analysis, the surfactant is initially added to the cuvette, followed by the addition of **DQCh** to ensure its complete solubility and stability in the medium. Lastly, an aliquot of white wine is added. The AOAC method for bisulfite determination was used to compare the results. As shown in Table 3, the recoveries were between 79.6 and 120.1%, showing that the present method can be potentially applied to the determination of bisulfite. Figure S9 shows how fluorescence intensity varies with an aliquot of white wine compared with an excess of bisulfite, exhibiting similar bands. The results obtained through our method are comparable to the AOAC method and are within the maximum margins allowed by the International Organization of Vine and Wine (OIV), which set a threshold of 200 mg L^–1^ for white wines.^73^ The developed method offers several advantages, including simplicity, one-step analysis, selectivity, sensitivity, and the requirement of only a minimal sample volume. Further studies have been performed to optimize the experimental conditions of the method to improve its accuracy. We aim to demonstrate the applicability of the method in different food samples in future studies, developing a complete analytical procedure. Each sample requires a specific pretreatment procedure to avoid interferences. In this work, the direct determination of bisulfite in white wine was feasible without any sample pretreatment.

**Table 3:** Determination of Bisulfite Concentration in Real Samples

sample	fluorometric method DQCh + CTABr (mg L^–1^)	AOAC method (mg L^–1^)^73^	recovery (%)
wine 1	145.9 ± 6.3	121.5 ± 4.2	120.1
wine 2	57.4 ± 1.1	72.0 ± 1.6	79.6
wine 3	120.9 ± 9.7	129.1 ± 2.3	93.6

## 4 Conclusions

In summary, a new probe based on 7-(diethylamino)­quinoline-2­(1*H*)-one chalcone (**DQCh**) was synthesized. We found that the probe changes its photophysical properties when exposed to an aqueous micellar solution of CTABr. On the other hand, the computational analysis allowed to predict that the electrophilic site in the probe was double bound “C–C” between the quinolin-2­(1*H*)-one and chalcone moieties, as confirmed by ^1^H NMR after the addition of bisulfite. Based on this finding, **DQCh** showed a significant selectivity toward bisulfite with a low LOD, sponsoring the use of a new fluorescent probe. Additionally, this new fluorescent probe was successfully applied to the detection of bisulfite in real food samples. These results support the idea that this type of probe in the presence of a micellar cationic medium may open the possibility of using this type of surfactant as a potential medium for future applications in analytical sensing.

## Supplementary Material



## Notes

Additional spectroscopic and optical characterization for **DQCh** and HSO_3_^–^•micelles and some examples of fluorescent probes of bisulfite (PDF)
