# Supplementary material for: An Efficient and Selective 7‑(Diethylamino)quinolin-2(1H)‑One-Chalcone Fluorescent Probe for Detecting Bisulfite in Wine Samples Using a Micellar Solution
Source: ACS Omega. 2025 Jun 17;10(25):26639–49. doi: 10.1021/acsomega.5c00828 (PMC12223884; doi:10.1021/acsomega.5c00828)
Supplement: Supplementary file 1 [file ao5c00828_si_001.pdf]

Supplementary Material

An efficient and Selective 7-(Diethylamino)quinolin-2(1*H*-)-one-chalcone Fluorescent Probe for Detecting Bisulfite in Wine Samples Using a Micellar Solution

Guillermo E. Quintero,[a] William Tiznado,[b] Luis Leyva-Parra,[c] Catalina Espinoza,[a] Oriel Sánchez-Velasco,[a] Edwin G. Pérez, [a] Carlos Rojas-Romo,[d] Christian Espinosa-Bustos [e] and Margarita E. Aliaga.*[,a]

[a] *Facultad de Química y de Farmacia, Escuela de Química, Pontificia Universidad Católica de Chile, Casilla 306, Santiago 6094411, Chile.*

[b] *Centro de Química Teórica & Computacional (CQT&C), Departamento de Ciencias Químicas, Facultad de Ciencias Exactas, Universidad Andrés Bello República 275, 8370146, Santiago, Chile.*

[c] *Centro de Investigación en Ingeniería de Materiales (CIIM), Facultad de Ingeniería y Arquitectura, Universidad Central de Chile (UCEN), Santa Isabel 1186, 8370146, Santiago, Chile.*

[d] *Departamento de Química, Facultad de Ciencias, Universidad de Chile, Las Palmeras 3425, Ñuñoa, Santiago 7800003, Chile.*

[e] *Facultad de Química y de Farmacia, Escuela de Química y de Farmacia, Pontificia Universidad Católica de Chile, Casilla 306, Santiago 6094411, Chile.*

Table of contents

Contents **Page**

Table S1. Summary of some fluorescent probe for bisulfite in wine samples. 3

Figure S1. ^{1}H-NMR spectra of (*E*)-7-(diethylamino)-3-(3-(4-fluorophenyl)-3-oxoprop-1-en-1-yl)-1-methylquinolin-2(1*H*)-one probe (**DQCh**) in ACN-d_3. 5

Figure S2. ^{13}C-NMR spectra of (*E*)-7-(diethylamino)-3-(3-(4-fluorophenyl)-3-oxoprop-1-en-1-yl)-1-methylquinolin-2(1*H*)-one probe (**DQCh**) in ACN-d_3. 6

Figure S3. ^{19}F-NMR spectra of (*E*)-7-(diethylamino)-3-(3-(4-fluorophenyl)-3-oxoprop-1-en-1-yl)-1-methylquinolin-2(1*H*)-one probe (**DQCh**) in ACN-d_3. 7

Figure S4. HR-MS of (*E*)-7-(diethylamino)-3-(3-(4-fluorophenyl)-3-oxoprop-1-en-1-yl)-1-methylquinolin-2(1*H*)-one probe (**DQCh**)..8

Figure S5. Steady-state fluorescence spectra of **DQCh** (1.8 μM) in various solutions of increasing polarity. Insert: The picture was taken under illumination with low-intensity light, λ = 345 nm, using a UV-A hand lamp. 9

Figure S6. Time-dependent (0 to 3 h) absorption spectrum of **DQCh** (1.8 μM) in the presence of 50 equiv. of HSO_3^- in SB3-14 (0.22 mM) and PBS (10 mM) at pH 7.4 and 25ºC. 10

Figure S7. ^{1}H-NMR spectrum of SB3-14 only and in the presence of bisulfite in D_2O. 10

Figure S8. ^{1}H-NMR spectrum of CTABr only and in the presence of bisulfite in D_2O. 11

Figure S9. Fluorescence spectrums of **DQCh** (1.8 μM) (▬), after of a white wine aliquot (▬) and a 50 equiv. of HSO_3^- (▬) at 27 ºC...11

Table S1. Summary of some fluorescent probe for bisulfite in wine samples.

Probe	Conditions	LOD (μM)	Lineal range (μM)	Ref.
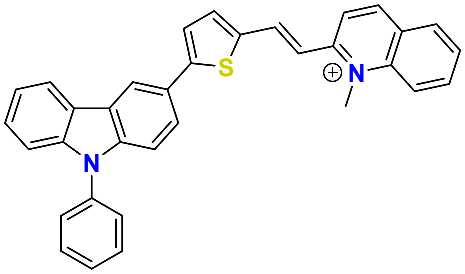	DMSO/PBS 3:7, pH 7.4, 1mM CTABr	0.072	0.24 - 20	1
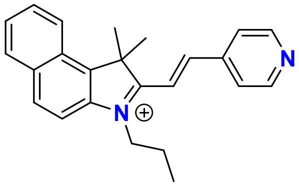	DMSO/PBS 1:99, pH 7.4	0.68	2.3 - 50	2
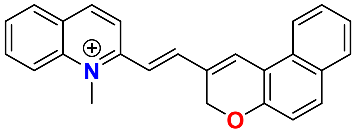	DMSO/PBS 2:98, pH 7.4, 1mM CTABr	2.1	7 - 16	3
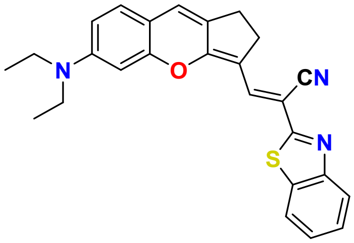	DMF/PBS 1:1, pH 8.4	9.48	25 - 170	4
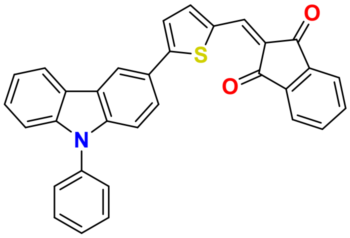	DMSO/PBS 3:7, pH 7.4, 1mM CTABr	0.058	0.19 - 12	5
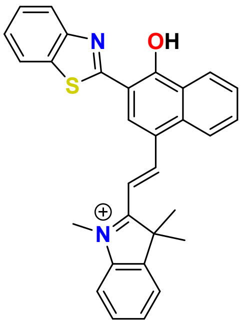	DMSO/PBS 7:3, pH 7.4	0.21	0.71 - 300	6
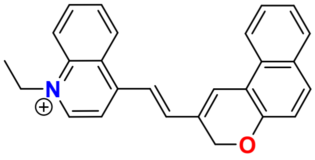	DMSO/PBS 2:98, pH 7.4, 1mM CTABr	1.20	4 - 25	7

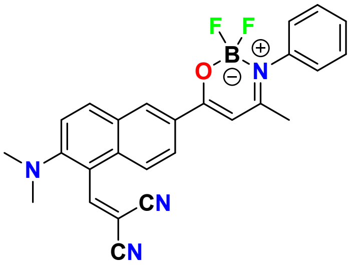	ACN/Tris buffer 85:15, pH 7.4	0.55	1.8 - 1000	8
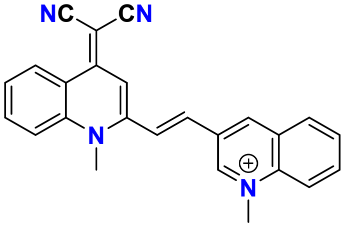	DMSO/PBS 3:7, pH 7.4	0.032	0.1 - 20	9
This Work	DMSO:PBS 1:99 pH 7.4 10mM CTABr	0.7	2.1 - 47.9	–

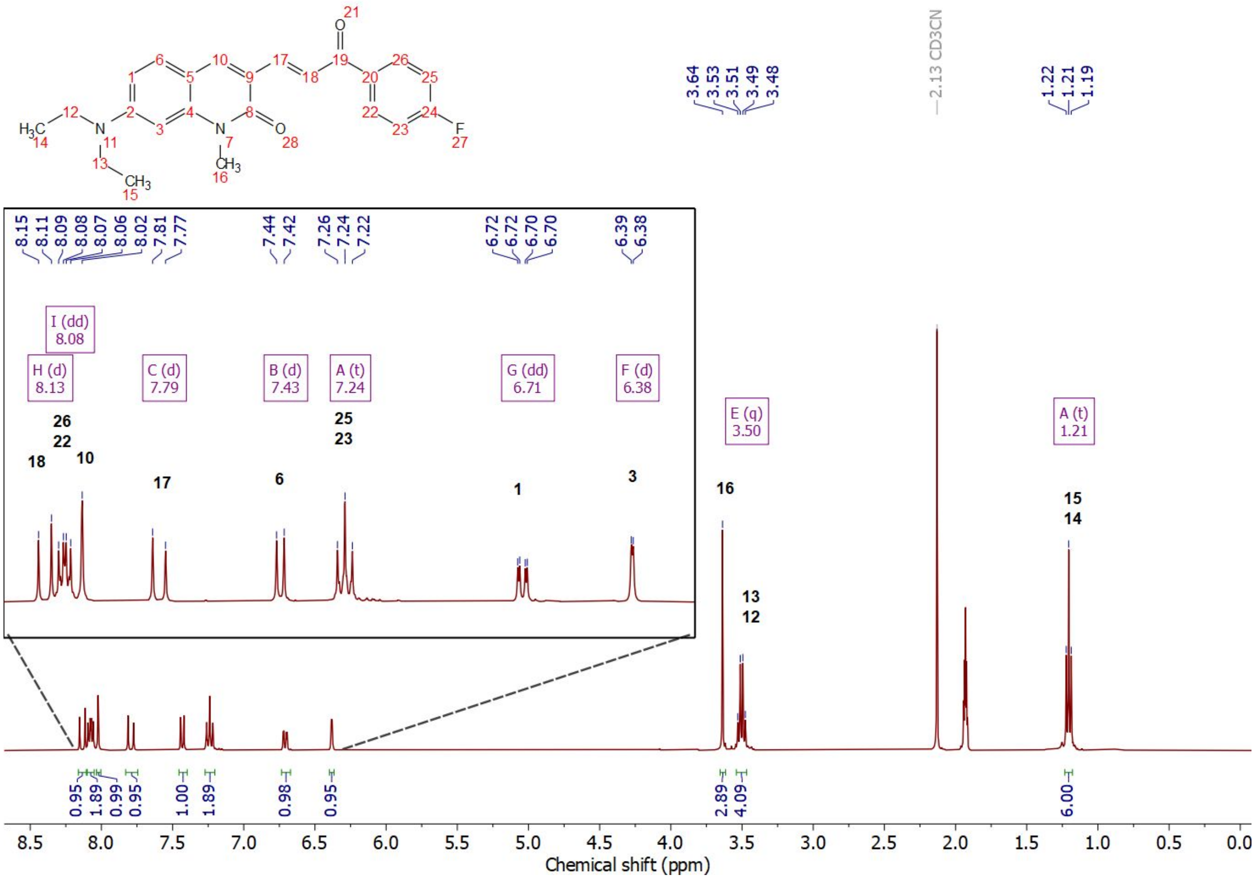

Figure S1. ^{1}H-NMR spectra of (*E*)-7-(diethylamino)-3-(3-(4-fluorophenyl)-3-oxoprop-1-en-1-yl)-1-methylquinolin-2(1*H*)-one probe (**DQCh**) in ACN-d_3.

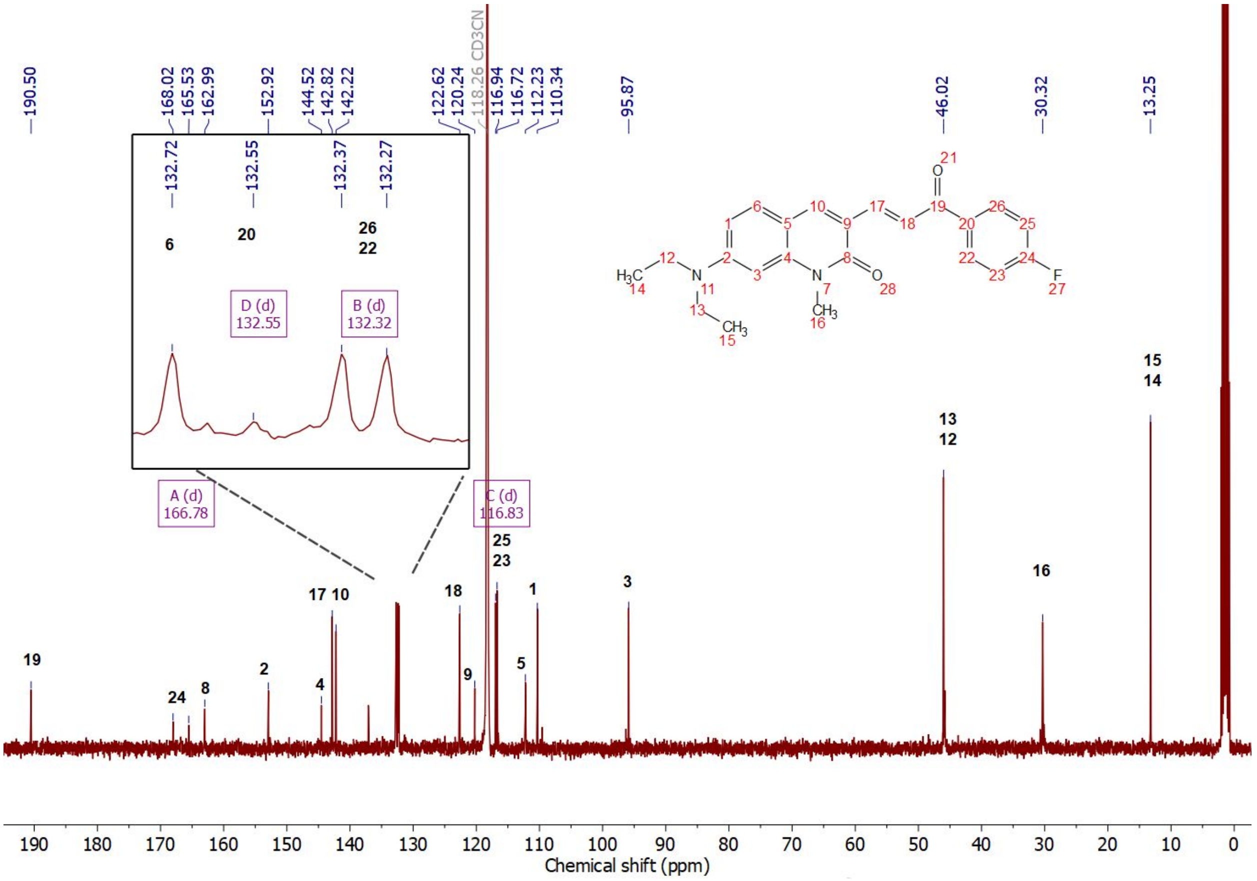

Figure S2. ^{13}C-NMR spectra of (*E*)-7-(diethylamino)-3-(3-(4-fluorophenyl)-3-oxoprop-1-en-1-yl)-1-methylquinolin-2(1*H*)-one probe (**DQCh**) in ACN-d_3.

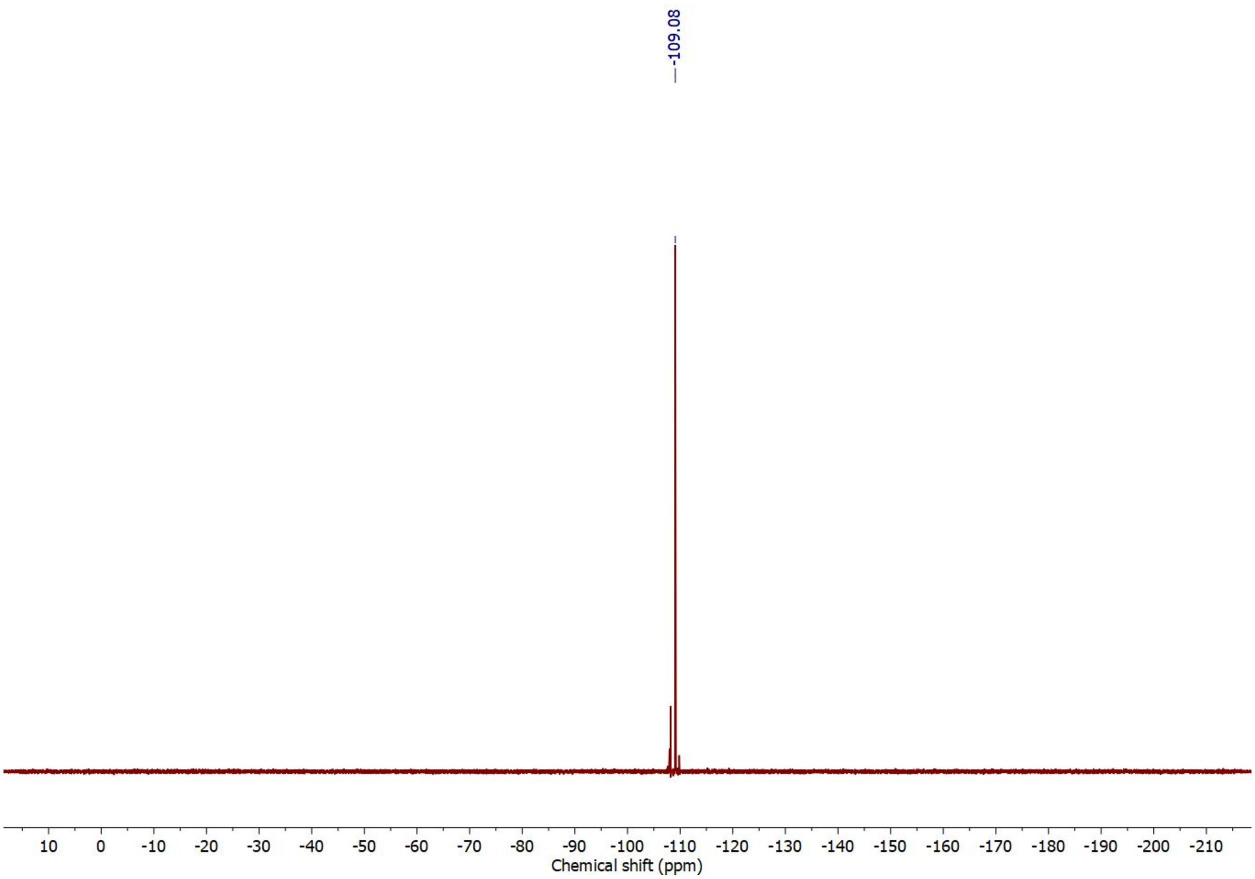

Figure S3. ^{19}F-NMR spectra of (*E*)-7-(diethylamino)-3-(3-(4-fluorophenyl)-3-oxoprop-1-en-1-yl)-1-methylquinolin-2(1*H*)-one probe (**DQCh**) in ACN-d_3.

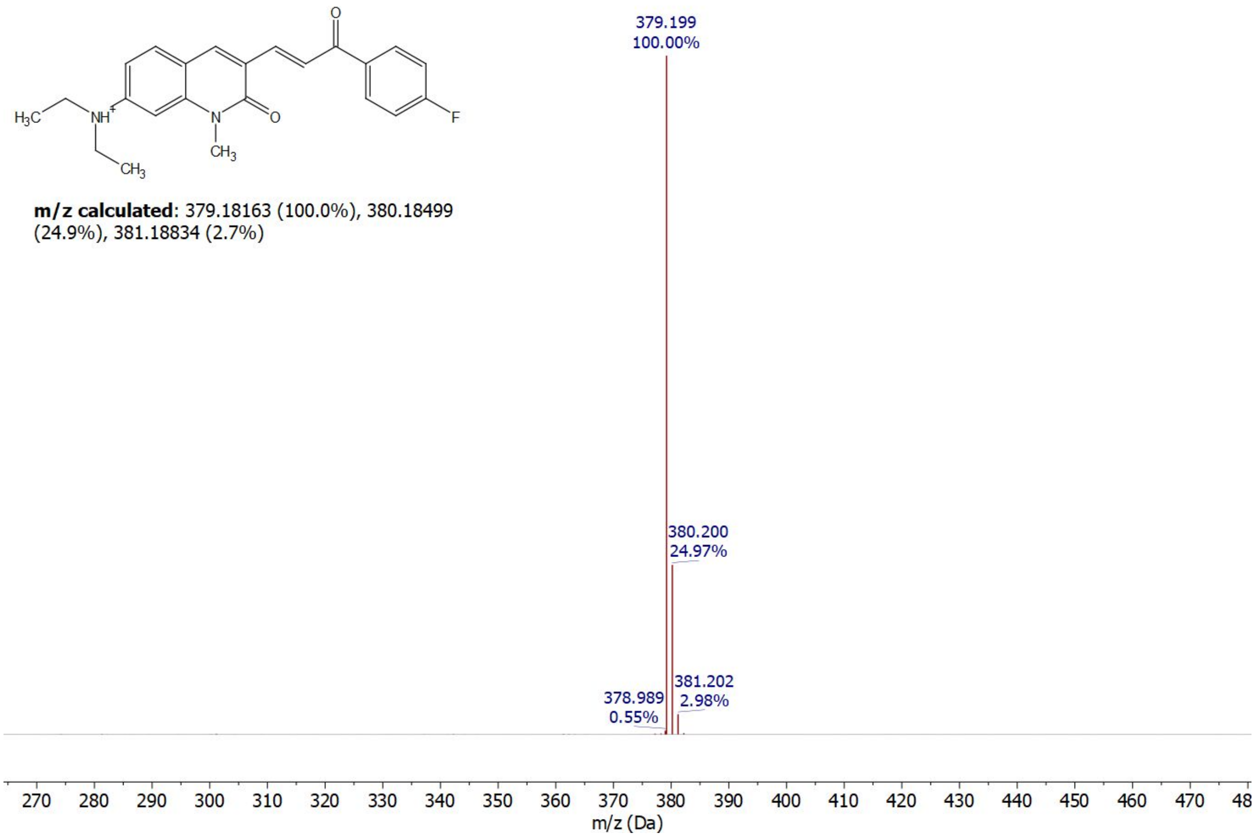

Figure S4. HR-MS of (*E*)-7-(diethylamino)-3-(3-(4-fluorophenyl)-3-oxoprop-1-en-1-yl)-1-methylquinolin-2(1*H*)-one probe (**DQCh**).

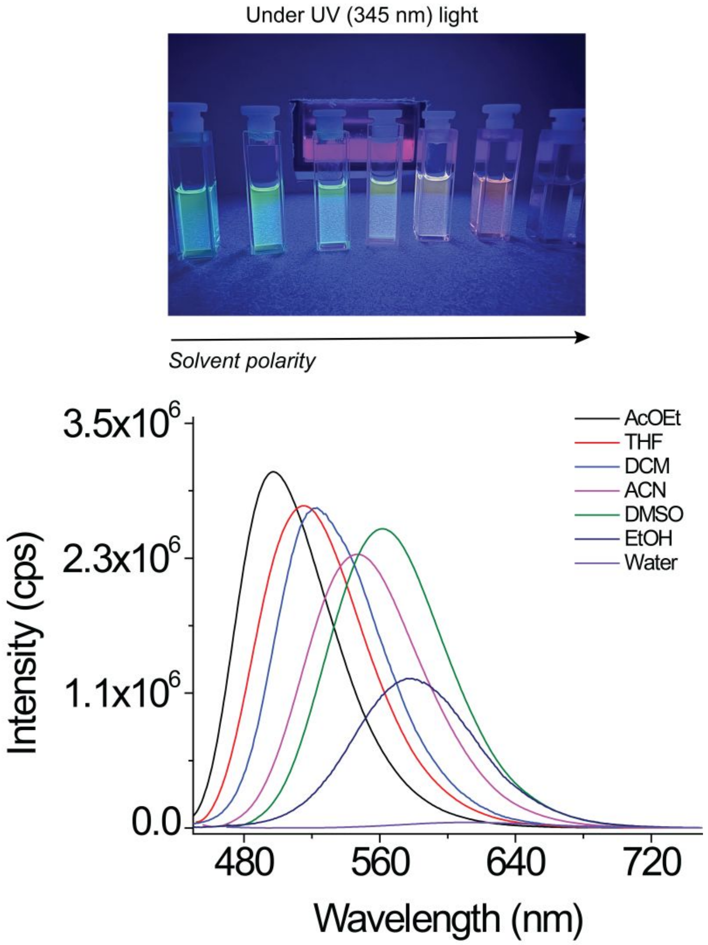

Figure S5. Steady-state fluorescence spectra of **DQCh** (1.8 μM) in various solutions of increasing polarity. Insert: The picture was taken under illumination with low-intensity light, λ = 345 nm, using a UV-A hand lamp.

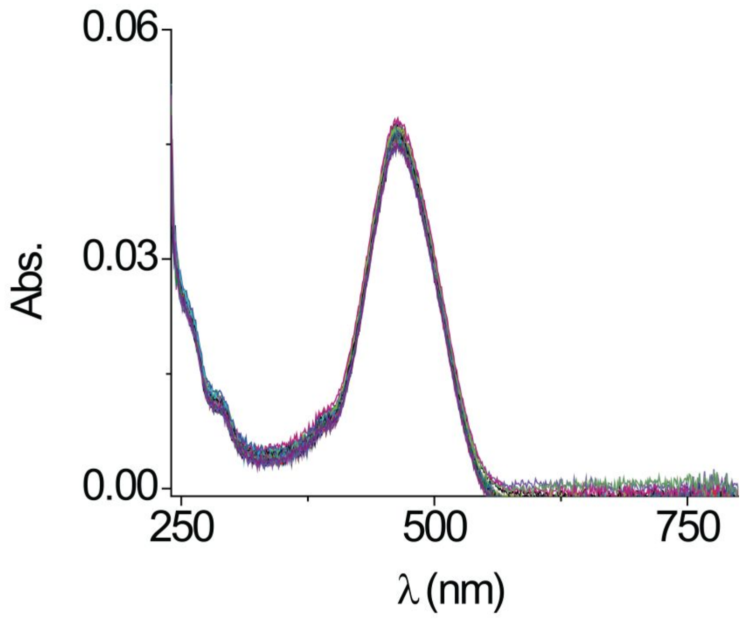

Figure S6. Time-dependent (0 to 3 h) absorption spectrum of **DQCh** (1.8 μM) in the presence of 50 equiv. of HSO_3^- in SB3-14 (0.22 mM) and PBS (10 mM) at pH 7.4 and 25ºC.

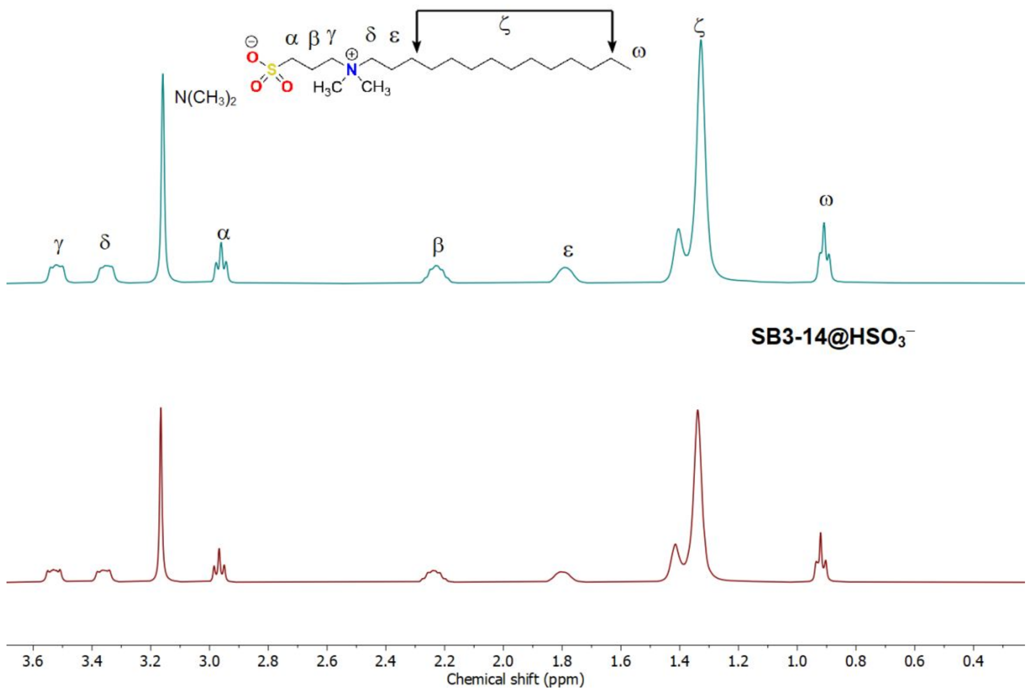

Figure S7. ^{1}H-NMR spectrum of SB3-14 only and in the presence of bisulfite in D_2O.

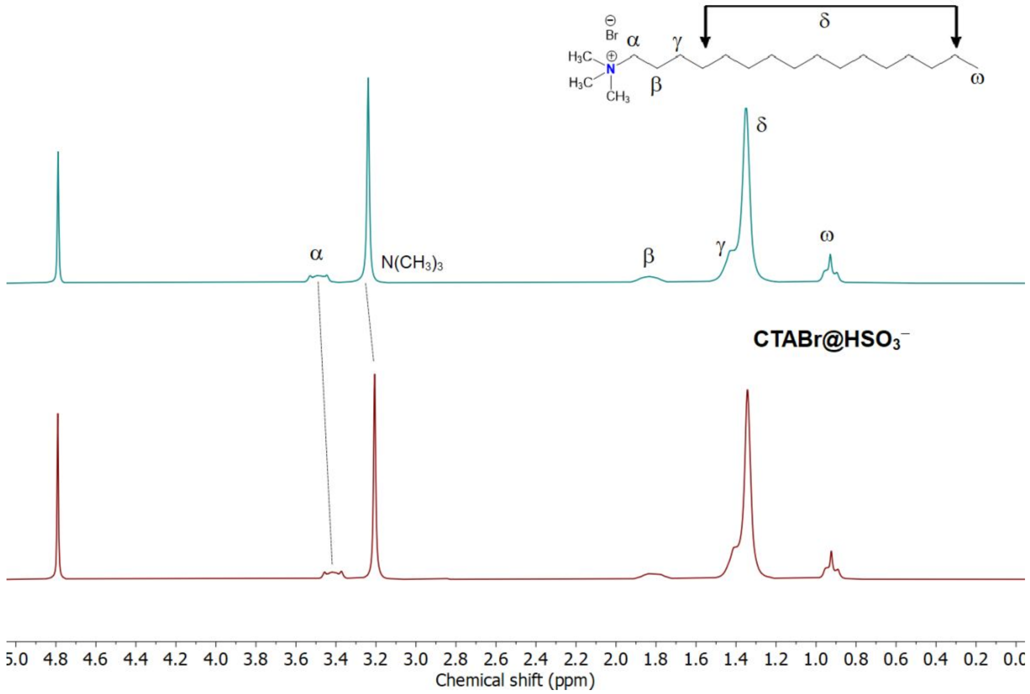

Figure S8. ^{1}H-NMR spectrum of CTABr only and in the presence of bisulfite in D_2O.

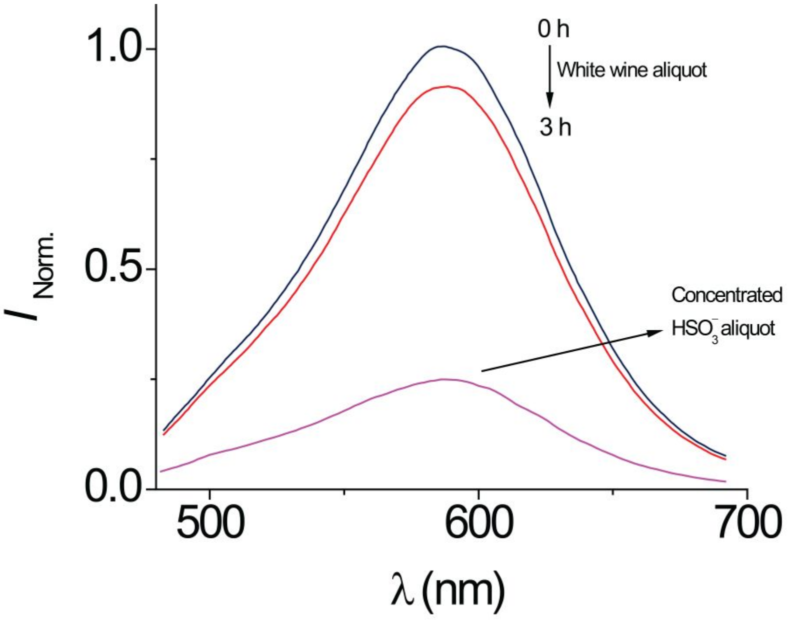

Figure S9. Fluorescence spectrums of **DQCh** (1.8 μM) (–), after of a white wine aliquot (–) and a 50 equiv. of HSO_3^- (–) at 27 ºC.

References

(1) Shang, Z.; Wang, Y.; Meng, Q.; Zhang, R.; Zhang, Z. A Near-Infrared Fluorescent Probe for Imaging of Bisulfite in Living Animals and Its Application in Food Samples. *Spectrochim Acta A Mol Biomol Spectrosc* 2023, *299*, 122853. https://doi.org/10.1016/j.saa.2023.122853.

(2) Zhang, D.; Wang, S.; Yang, F.; Li, Z.; Huang, W. Visual Inspection of Acidic PH and Bisulfite in White Wine Using a Colorimetric and Fluorescent Probe. *Food Chem* 2023, *408*, 135200. https://doi.org/10.1016/j.foodchem.2022.135200.

(3) Shang, Z.; Liu, J.; Hu, Z.; Meng, Q.; Wang, Y.; Zhang, R.; Zhang, Z. A Near-Infrared Fluorescence Probe for the Detection of Bisulfite in Vivo and Food Samples. *Dyes and Pigments* 2022, *200*, 110119. https://doi.org/10.1016/j.dyepig.2022.110119.

(4) Li, Y.; Sun, X.; Zhou, L.; Tian, L.; Zhong, K.; Zhang, J.; Yan, X.; Tang, L. Novel Colorimetric and NIR Fluorescent Probe for Bisulfite/Sulfite Detection in Food and Water Samples and Living Cells Based on the PET Mechanism. *J Agric Food Chem* 2022, *70* (35), 10899–10906. https://doi.org/10.1021/acs.jafc.2c04571.

(5) Shang, Z.; Liu, J.; Meng, Q.; Jia, H.; Gao, Y.; Zhang, C.; Zhang, R.; Zhang, Z. Carbazole-Based near-Infrared-Emitting Fluorescence Probe for the Detection of Bisulfite in Live Animals and Real Food Samples. *New Journal of Chemistry* 2022, *46* (43), 20737–20744. https://doi.org/10.1039/D2NJ04647D.

(6) Qin, J.; Kong, F.; Guo, Y.; Wang, D.; Zhang, C.; Li, Y. Rational Construction of a Two-Photon NIR Ratiometric Fluorescent Probe for the Detection of Bisulfite in Live Cells, Tissues, and Foods. *J Agric Food Chem* 2022, *70* (23), 7314–7320. https://doi.org/10.1021/acs.jafc.2c02155.

(7) Shang, Z.; Liu, J.; Meng, Q.; Wang, Y.; Zhang, C.; Zhang, Z. A Near-Infrared Emitted Fluorescence Probe for the Detection of Biosulfite in Live Zebrafish, Mouse and Real Food Samples. *Methods* 2022, *204*, 47–54. https://doi.org/10.1016/j.ymeth.2022.04.007.

(8) Su, C.-C.; Kim, K.-R.; Hong, J.-I. Dual-Functional Turn-on Fluorescent Probe for Discriminative Sulfite and Sulfide Detection via Organic/Aqueous Ratio Tuning and Its Application in Real Samples. *Dyes and Pigments* 2022, *206*, 110669. https://doi.org/10.1016/j.dyepig.2022.110669.

(9) Yue, C.; Zeng, L.; Zhang, D.; Li, K.; Jiang, L.; Xie, P. A Practical Chromogenic and Fluorogenic Dual-Mode Sensing Platform for Rapid Quantification of Sulfite in Food. *Food Chem* 2024, *440*, 138183. https://doi.org/10.1016/j.foodchem.2023.138183.
